# Growth-Factor-Driven Rescue to Receptor Tyrosine Kinase (RTK) Inhibitors through Akt and Erk Phosphorylation in Pediatric Low Grade Astrocytoma and Ependymoma

**DOI:** 10.1371/journal.pone.0122555

**Published:** 2015-03-23

**Authors:** Mariska Sie, Wilfred F. A. den Dunnen, Harm Jan Lourens, Tiny G. J. Meeuwsen-de Boer, Frank J. G. Scherpen, Walderik W. Zomerman, Kim R. Kampen, Eelco W. Hoving, Eveline S. J. M. de Bont

**Affiliations:** 1 Department of Pediatrics, Beatrix Children’s Hospital, Pediatric Oncology/Hematology division, University Medical Center Groningen, University of Groningen, Groningen, the Netherlands; 2 Department of Pathology and Medical Biology, Pathology division, University Medical Center Groningen, University of Groningen, Groningen, the Netherlands; 3 Department of Neurosurgery, University Medical Center Groningen, University of Groningen, Groningen, the Netherlands; Swedish Neuroscience Institute, UNITED STATES

## Abstract

Up to now, several clinical studies have been started investigating the relevance of receptor tyrosine kinase (RTK) inhibitors upon progression free survival in various pediatric brain tumors. However, single targeted kinase inhibition failed, possibly due to tumor resistance mechanisms. The present study will extend our previous observations that vascular endothelial growth factor receptor (VEGFR)-2, platelet derived growth factor receptor (PDGFR)β, Src, the epidermal growth factor receptor (ErbB) family, and hepatocyte growth factor receptor (HGFR/cMet) are potentially drugable targets in pediatric low grade astrocytoma and ependymoma with investigations concerning growth-factor-driven rescue. This was investigated in pediatric low grade astrocytoma and ependymoma cell lines treated with receptor tyrosine kinase (RTK) inhibitors *e*.*g*. sorafenib, dasatinib, canertinib and crizotinib. Flow cytometry analyses showed high percentage of cells expressing VEGFR-1, fibroblast growth factor receptor (FGFR)-1, ErbB1/EGFR, HGFR and recepteur d’origine nantais (RON) (respectively 52-77%, 34-51%, 63-90%, 83-98%, 65-95%). Their respective inhibitors induced decrease of cell viability, measured with WST-1 cell viability assays. At least this was partially due to increased apoptotic levels measured by Annexin V/Propidium Iodide apoptosis assays. EGF, HGF and FGF, which are normally expressed in brain (tumor) tissue, showed to be effective rescue inducing growth factors resulting in increased cell survival especially during treatment with dasatinib (complete rescue) or sorafenib (partial rescue). Growth-factor-driven rescue was less prominent when canertinib or crizotinib were used. Rescue was underscored by significantly activating downstream Akt and/or Erk phosphorylation and increased tumor cell migration. Combination treatment showed to be able to overcome the growth-factor-driven rescue. In conclusion, our study highlights the extensive importance of environmentally present growth factors in developing tumor escape towards RTK inhibitors in pediatric low grade astrocytoma and ependymoma. It is of great interest to anticipate upon these results for the design of new therapeutic trials with RTK inhibitors in these pediatric brain tumors.

## Introduction

**Fig 1:**
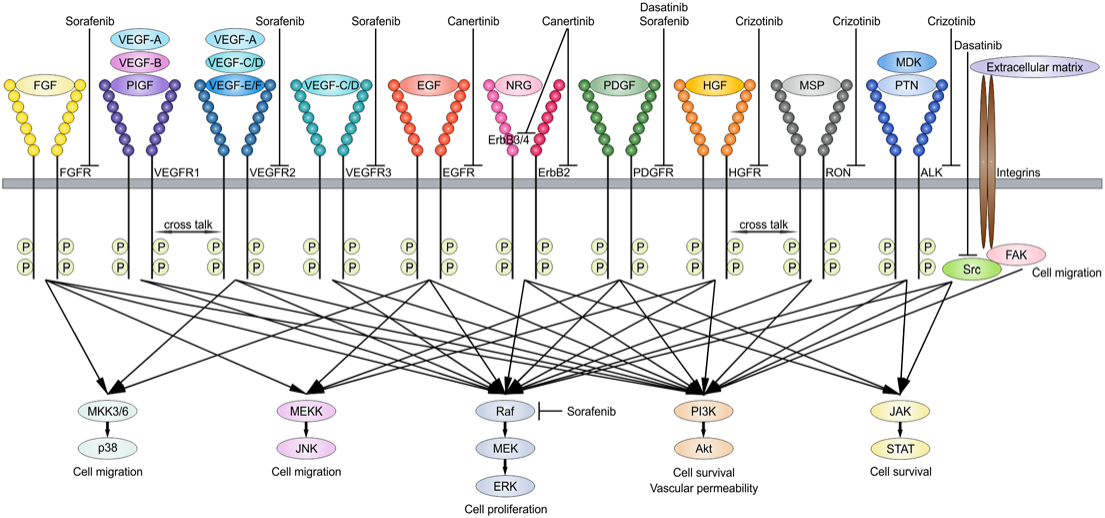
RTK signaling pathways. Schematic illustration showing important growth factors in brain tumors that can bind RTKs activating downstream pathways which contributes to tumor cell survival. These potential drugable targets can be inhibited by *e*.*g*. sorafenib, dasatinib, canertinib and crizotinib.

Low grade astrocytomas (WHO grade I-II) are the most frequent brain tumors in children. Ependymoma (WHO grade II-III) accounts for 6–12% of all pediatric brain tumors and is the fourth most common brain tumor in children, after low grade astrocytoma, medulloblastoma (WHO grade IV) and high grade astrocytoma (WHO grade III-IV).[1] Although the 5-year survival of patients with pilocytic astrocytoma (WHO grade I) is around 90%, morbidity can be serious mainly because of the tumor localization and the change of surgical morbidity.[2,3] Moreover, despite developments in neurosurgery, chemotherapy and radiation, the 5-year survival of pediatric ependymoma is approximately 57%.[4] Therefore, a search for new targeted therapies has started. With kinome profiling we previously identified vascular endothelial growth factor receptor 2 (VEGFR-2), platelet derived growth factor receptor β (PDGFRβ), Src, the epidermal growth factor receptor family (ErbB1-4), and hepatocyte growth factor receptor (HGFR/cMet) as potentially drugable targets in these pediatric brain tumors.[5] Potential interesting inhibitors for these targets are sorafenib, dasatinib, canertinib and crizotinib respectively (overview in Fig. 1). Today, still very limited data is published about the clinical use of inhibitors targeting these receptor tyrosine kinases (RTKs) in pediatric brain tumors, and even less is known in low grade astrocytoma and ependymoma.

Up to now, mainly pediatric high grade astrocytomas and just a few ependymomas were included in phase I studies analyzing erlotinib, an ErbB1/EGFR TK inhibitor as a single agent and in combination with chemotherapy or radiation.[6–8] Erlotinib was well tolerated in children, as were other ErbB family inhibitors including gefitinib and lapatinib.[6–10] The only published phase II study showed unfortunately no increase in progression free survival or overall survival with gefitinib and radiation in malignant pediatric brain tumors.[10] Currently, erlotinib is under investigation in pediatric low grade astrocytoma and ependymoma in phase I and II trials respectively. As the ErbB TK family comprises four members, canertinib, a new pan-ErbB TK inhibitor showing anti-proliferative and pro-apoptotic effects on tumor cells,[11] could be more interesting. Canertinib has not been investigated in pre- or clinical pediatric brain tumor studies. Sorafenib has been described in clinical trials, yet only restricted to adult brain tumors. Limited activity was reported of sorafenib in recurrent glioblastoma and in the first-line therapy for glioblastoma.[12–14] Recently, sorafenib, crizotinib and dasatinib have been introduced in pediatric brain tumors.[15,16] Overall, the preliminary results of single receptor tyrosine kinase targeted tumor therapies are disappointing.

Although in chronic myeloid leukemia, single kinase targeted therapy, for oncogenic activated BCR/Abl has proven very successful,[17] more recent trials in other malignancies failed to show prolonged responses. It is thought that tumor progression is the net result of signaling through various protein kinase mediated networks driving tumor cell proliferation and survival. The signaling networks can be reflected by oncogenic mutations, silence tumor suppressor mutations, epigenetic changes and stromal interaction and crosstalk, such as in angiogenesis. Important tumor related growth factors which are normally expressed in the developing brain binding RTKs include VEGF, EGF, HGF, FGF and PDGF. Altogether these changes result in specific signaling profiles which promote tumor cell growth (Fig. 1).

In various tumor types, it has been shown that single targeted kinase inhibition failed due to tumor escape mechanisms through alternate routes of kinase pathway activation.[18] For example, RTK upregulation has been observed following targeted inhibition of selective kinases. This kinome reprogramming circumvents inhibition of proto-oncogenic kinases as for instance described for oncogenic RAS.[19] Furthermore, inhibition of specific RTKs could trigger the tumor to upregulate RTK ligands.[20,21] An increase in RTK ligands through autocrine tumor-cell production, paracrine contribution from tumor stroma or systemic production, could eventually contribute to tumor resistance to the RTK inhibitor with a similar signaling output as shown recently by Wilson et al in oncogenic mutated cell lines.[22] The moderate preliminary results of single RTK inhibitors in pediatric brain tumors could possibly be due to resistance mechanisms. The present study will extend our previous observations with investigations concerning growth-factor-driven rescue in pediatric low grade astrocytoma and ependymoma treated with RTK inhibitors *e*.*g*. sorafenib, dasatinib, canertinib and crizotinib.

## Materials and Methods

### Cell cultures

Three pediatric low grade astrocytoma cell lines (WHO grade I: Res-186, grade II: Res-259 and UW-467) and one ependymoma cell line (Res-196) were evaluated. These cell lines were kindly given by Dr. Michael S. Bobola (Seattle Children’s Hospital Research Institute, USA).[23] All cell lines were cultured in DMEM/F12 (Life Technologies, Carlsbad, CA, USA) containing 5% fetal calf serum (FCS). Cell cultures contained 100 units/ml penicillin and 100 ul/ml streptomycin (PAA Laboratories, Les Mureaux, France).

### Flow cytometry analyses

Cells were blocked by PBS 1% BSA (Bovine Serum Albumin, Sigma Aldrich, St Louis, MO, USA), and stained with anti-VEGFR-1 (Sigma Aldrich), anti-VEGFR-2-PE (Sigma Aldrich), anti-VEGFR-3-APC (R&D Systems, Minneapolis, MN, USA), anti-EGFR (Abcam, Cambridge, UK), anti-ErbB2-PE (R&D Systems), anti-ErbB3-APC (R&D Systems), anti-ErbB4 (R&D Systems), anti-HGFR/cMet-FITC (R&D Systems), anti-RON-APC (R&D Systems), anti-FGFR-1 (Cell Signaling, Danvers, MA, USA), anti-FGFR-2 (R&D Systems), anti-PDGFRα-biotin (BD Biosciences, San Jose, CA, USA), anti-PDGFRβ-PE (BD Biosciences). Primary FGFR-2, ErbB4 antibodies were visualized using a Rabbit anti-Mouse PE-conjugated secondary antibody (Dako Cytomation, Glostrup, Denmark), and FGFR-1 with a FITC-conjugated Swine anti-Rabbit secondary antibody (Dako Cytomation). Primary EGFR was visualized using Alexa Fluor 488 conjugated anti-Rat secondary antibody (Cell Signaling) and PDGFRα antibody was visualized using streptavidin FITC (BD Biosciences). IgG-FITC/PE/APC and secondary antibodies were used as negative (isotype) controls. Expression was analyzed using LSRII (BD FACS DIVA software, BD Biosciences). The data was eventually developed using FlowJo software (Tree Star Inc., Ashland, OR, USA). Expression levels above 5% were considered as actual membrane protein expression, reaching above isotype controls.

### Human growth factor antibody array

Human growth factor antibody array membranes (Abcam) were used to measure growth factor protein profile of the used low grade astrocytoma and ependymoma cell lines following manufacturer’s protocol. Each protein specific antibody is spotted in duplicate on a nitrocellulose membrane. Equal amounts of proteins were applied to the array. Levels of proteins were assessed using a horseradish peroxidase (HRP-)conjugated Streptavidin followed by chemiluminescence detection. Signal intensities were analyzed using ScanAlyze array software (Eisen Lab Software). Normalized signal intensity was calculated with the formula: X(y) x P1/P(y). P1 = mean signal density—background of positive control spots on culture medium array used as reference array, P(y) = mean signal density—background of positive control spots on array of specific cell line, X(y) = mean signal density—background for spot X (protein of interest) on array for sample y (specific cell line).

### Cell viability assays

Crystal violet staining was used to analyze the effect of growth factors on the different pediatric low grade astrocytoma and ependymoma cells. Furthermore, WST-1 colorimetric viability assays were performed for studying effects of RTK inhibitors and growth factors on tumor cell viability following the protocol described by the manufacturer (Roche, Basel, Switzerland). Cells were seeded in 96-wells plates at a density of 15 x 10^3^ per well in medium (1% FCS). After cell adhesion, growth factors including VEGF-A, EGF human, HGF human, FGF basic, PDGF-AB (all 100 ng/ml, Life Technologies)[22,24] were added. To determine single effects of these growth factors on tumor cell count after 48h, cells were fixed with 4% formaldehyde. After 20 minutes, cells were washed followed by incubation with 0.04% crystal violet in 4% ethanol for 30 minutes. Then again cell washing, air dried and incubated with 1% SDS solution on a shaker for 1h. Optical density was measured at 595 nm in a microplate reader (Bio-Rad Laboratories, Hercules, CA, USA) and tumor cell count was calculated using an internal calibrator.

During WST cell viability assays, tumor cells were treated after adhesion with sorafenib (0–8 uM), dasatinib (0–1000 nM), canertinib (0–10 uM) or crizotinib (0–12 uM) for 48h, in which the doses were based on literature.[5,25–30] In the combination treatments, the second inhibitor was added at the following concentrations: sorafenib 2 uM, canertinib 4 uM or crizotinib 3 uM. Sorafenib, dasatinib, canertinib and crizotinib (LC laboratories, Woburn MA, USA) were dissolved in sterile dimethyl sulfoxide (DMSO) and stored at -20°C. Similar growth factors as described previously were added 1h after incubation by the inhibitor. After addition of the WST-1 cell survival reagent the absorbance was measured at 450 nm in a microplate reader (Bio-Rad Laboratories). In every assay, per growth factor or inhibitor, each concentration contains equal concentrations of DMSO. For each concentration 6 replicates were included. Similar conditions were maintained for different combination treatments. Growth-factor-driven rescue during RTK inhibition was calculated with the formula: ((A = LC50 of cells treated with inhibitor plus growth factor)–(B = LC50 of the inhibitor-treated cells only)) / B x 100% if (A—standard deviation (SD))–(B + SD) > 0, otherwise it was defined as ‘no rescue’. If addition of the growth factor did affect the LC50 during RTK inhibition, this was marked as ‘partial rescue’. Complete rescue was noted as A > 10x B.[22]

### Apoptosis assays

For detection of (early) apoptotic and necrotic cells, Annexin V-FITC and Propidium Iodide (PI) were used. Cells were seeded in 6-wells plates at a density of 4.5 x 10^5^ per well in medium (1% FCS). After adhesion, sorafenib (0–8 uM), dasatinib (0–800 nM), canertinib (0–10 uM), or crizotinib (0–12 uM, LC laboratories) were added. In the combination treatments, the second inhibitor was added at the following concentrations: sorafenib 2 uM, canertinib 4 uM or crizotinib 3 uM. 1h after incubation of the inhibitor(s) growth factors including VEGF-A, EGF, HGF, FGF or PDGF (100 ng/ml, Life Technologies) were added. After 48h, cells were harvested and stained with Annexin V-FITC and PI according to the manufacturer's protocol. Quantitative analysis was conducted by flow cytometry. The data was eventually developed using FlowJo software (Tree Star Inc).

### Cell migration assays

Cell migration was analyzed using a scratch test. Cells were seeded in 6-wells plates at a density of 4.5 x 10^5^ per well in medium (1% FCS). Following adhesion, medium was removed and a horizontal ‘scratch’ was made in the cell monolayer using a 200-ul pipette tip. Then again medium (1% FCS) was added. Cells were incubated with sorafenib (0–1 uM) ± canertinib (4 uM), canertinib (0–2 uM) ± crizotinib (3 uM) or ± sorafenib (2 uM, LC laboratories). After 1h, growth factors including EGF, HGF or FGF (100 ng/ml, Life Technologies) were added. Cell migration into the scratched area was observed and imaged after 24h and 48h. Medium was refreshed directly before imaging at the later time point.

### Western blot analyses

1 x 10^6^ cells were seeded in T25 flasks in 5 ml DMEM-F12 containing 1% FCS to which after adhesion sorafenib 8 uM, dasatinib 1 uM, canertinib 8 uM or crizotinib 9 uM (LC laboratories) was added. In the combination treatments, the second inhibitor was added at a lower concentration: sorafenib 2 uM, canertinib 4 uM or crizotinib 3 uM. After 1h, growth factors including EGF, HGF, FGF or PDGF (all 100 ng/ml, Life Technologies) were added for another 1h. Cells were lysed in laemmli sample buffer (Bio-Rad Laboratories). Proteins were separated by sodium dodecyl sulfate—polyacrylamide gel electrophoresis (SDS-PAGE), and transported to nitrocellulose membranes. First, the membranes were incubated overnight with monoclonal primary antibodies for phospho-Erk (pErk) and total Erk (tErk), pAkt and tAkt, actin (Cell Signaling), followed by 1 hour incubation with HRP conjugated secondary antibodies (Dako Cytomation). Protein bands were visualized by chemiluminescence, on an x-ray film. β-actin was used as loading control. Normalized protein expression was calculated by dividing the signal intensity of the target protein by signal intensity of β-actin per sample using Image Studio Lite 4.0 software. Data was presented as normalized protein expression based on three independent experiments.

### Statistical analyses

Statistical comparisons within similar experiments were made with the non-parametric Wilcoxon signed-rank test. The Spearman correlation test was used to compute correlations between different techniques. For all statistical analyses, a two-tailed p value of less than 0.05 was considered significant.

## Results

### EGF, HGF and FGF driven rescue to various RTK inhibitors

**Fig 2:**
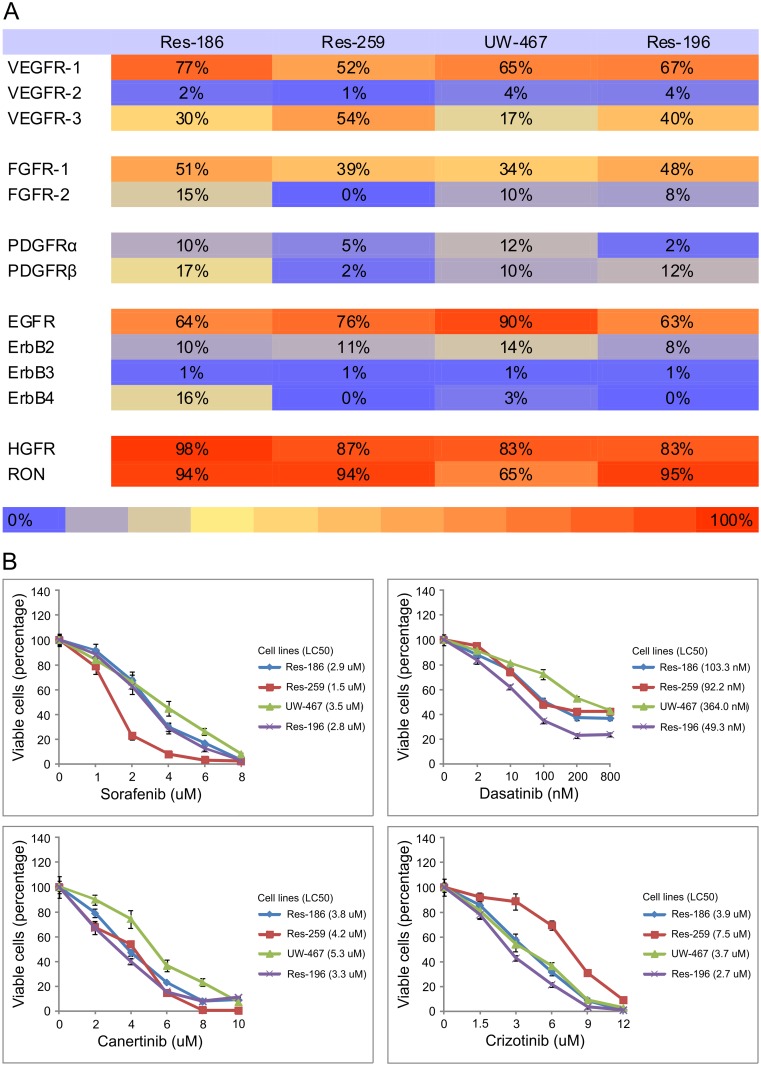
RTK expression and inhibition. **A**, Heat map showing percentages of cells expressing numerous growth factor receptors above control levels using flow cytometry analyses in pediatric low grade astrocytoma (Res-186, Res-259, UW-467) and ependymoma (Res-196) cell lines. **B**, Cell viability assays demonstrating the mean effect ± SD of sorafenib, dasatinib, canertinib and crizotinib on pediatric low grade astrocytoma (Res-186, Res-259, UW-467) and ependymoma (Res-196) cell lines after 48h.

Previously, with kinome profiling we showed PDGFRβ, Src, ErbB family members, and HGFR are highly activated in pediatric low grade astrocytoma and ependymoma and were found to be potentially drugable targets in vitro.[5] However, more recent clinical trials analyzing single targeted therapy in pediatric brain tumors show disappointing results, possibly due to tumor escape mechanisms when specific targets are inhibited. We started with flow cytometry analyses of RTK expression. Pediatric low grade astrocytoma and ependymoma cell lines showed high percentages of cells expressing VEGFR-1, FGFR-1, EGFR, HGFR and RON (respectively 52–77%, 34–51%, 63–90%, 83–98%, 65–95%, compared to isotype controls, Fig. 2A). Their inhibitors (sorafenib, dasatinib, canertinib and crizotinib respectively) showed decreased tumor cell viability (Fig. 2B), mainly a result of tumor cell apoptosis (data not shown). As expected canertinib and crizotinib induced lower phosphorylation levels of phosphorylated EGFR and HGFR respectively by western blotting (data not shown).

**Fig 3:**
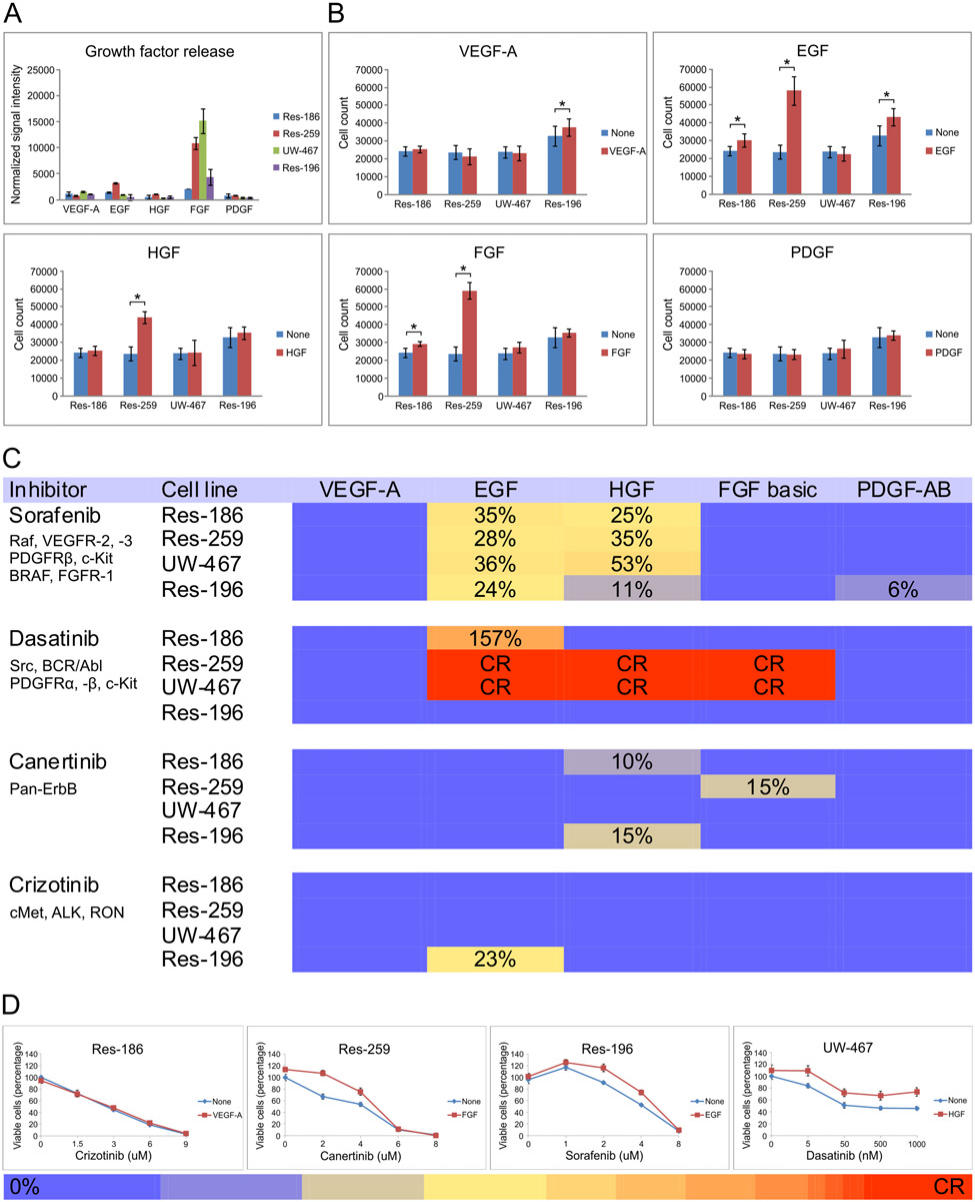
Growth-factor-driven rescue to RTK inhibitors. **A**, Human growth factor antibody array showing overall low growth factor release by pediatric low grade astrocytoma and ependymoma cell lines (mean ± SD), except for FGF. **B**, Cell counting assays showing mean effect (± SD) of growth factors on the different tumor cell lines in the absence of inhibitors (*p < 0.05). **C**, Heat map showing the effect of growth factors on tumor cells treated with different TKI’s. Growth-factor-driven rescue during RTK inhibition was calculated with the formula: ((A = LC50 of cells treated with inhibitor plus growth factor)–(B = LC50 of the inhibitor-treated cells only)) / B x 100% if (A—SD)–(B + SD) > 0, otherwise it was defined as ‘no rescue’ (purple squares). If addition of the growth factor did affect the LC50 during RTK inhibition, this was marked as ‘partial rescue’ (yellow/orange squares). Complete rescue was noted as A > 10x B (red squares). As an internal negative control no growth-factor-driven rescue was found of the particular RTK ligand of which the receptor is inhibited. **D**, Cell viability assays demonstrating various effects of growth factors on TKI-treated cells (mean ± SD), with a corresponding colored bar indicating the level of growth-factor-driven rescue.

The growth factors, VEGF, FGF, PDGF, EGF and HGF related to the abovementioned RTKs are normally expressed during brain development. Moreover high expression levels of these growth factors are previously found in brain tumor bulk. It is important to take into account that these results of crude tissue might have no relation with availability on cellular level where mechanisms of bound and soluble growth factors play an essential role related to specific stromal crosstalk. We assessed growth factor production by our cultured tumor cells themselves and showed in Fig. 3A that the overall release by tumor cells was low except for FGF. Therefore, we decided to continue these experiments by using consequent identical levels of growth factors as has been published previously.[24] Addition of different growth factors to the cell cultures without inhibitor showed that EGF, HGF and FGF increased cell numbers up to 2.5 times in Res-259 cells (Fig. 3B). Interestingly, inhibition by sorafenib, canertinib and crizotinib could be rescued partially, and even completely in dasatinib treated tumor cells (Fig. 3C and D). Especially EGF, HGF and FGF showed a strong rescue potential. Expression levels of the RTKs were not correlated with growth-factor-driven rescue in terms of increase in LC50.

### Growth factors overcome RTK-inhibited-phosphorylation of downstream targets

**Fig 4:**
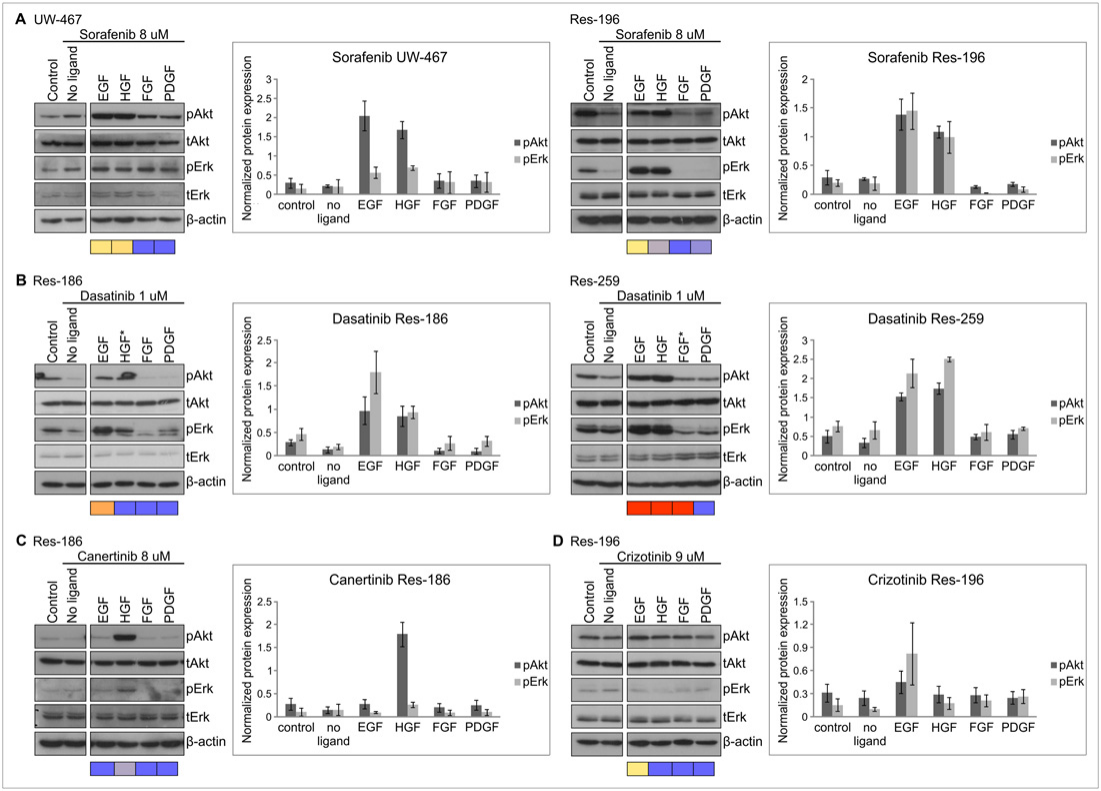
Growth factors overcome RTK-inhibited-phosphorylation of downstream targets. Examples of immunoblots showing effects of growth factors (100 ng/ml) on Akt and Erk phosphorylation (p) after sorafenib (**A**), dasatinib (**B**), canertinib (**C**), crizotinib (**D**) in pediatric low grade astrocytoma and ependymoma cells (2h). Controls represent the specific cell lines treated with DMSO. Growth-factor-driven rescue on cell viability as given in Fig. 3C and D is indicated underneath the blots: purple squares, no rescue; yellow/orange squares, partial rescue; red squares, complete rescue. Protein expression was normalized to β-actin as loading control and derived from three independent experiments of which the mean (± SEM) was calculated. EGF and HGF-driven rescue resulted in significantly higher protein expression of Akt (p = 0.028) and Erk (p = 0.043) during sorafenib in both UW-467 and Res-196 cells and during dasatinib in the low grade astrocytoma cell lines (p = 0.028 and p = 0.043). Significantly higher Akt phosphorylation was found after HGF addition during canertinib in Res-186, Res-259 and Res-196 cells (p = 0.043) of which Res-186 is shown as an example. *Discrepancies between growth-factor-driven rescue on cell viability level and downstream phosphorylation status.

We continued by assessing growth-factor-driven rescue on RTK-inhibited-phosphorylation of downstream targets to validate the previous rescue results on cell viability level. Analyses of the PI3K/Akt and MAPK/Erk downstream pathways, as the most commonly used survival signaling pathways by RTKs (Fig. 1), indeed underscored EGF- and HGF-driven rescue during sorafenib treatment which resulted in phosphorylation of Akt and Erk in all cell lines (UW-467 and Res-196 are shown as examples in Fig. 4A). Furthermore, complete rescue of EGF and HGF during dasatinib treatment resulted in significant Akt and Erk phosphorylation in Res-186 and Res-259 cells (Fig. 4B, UW-467 not shown). HGF-driven rescue, positively effecting cell viability during canertinib, was also confirmed with overcoming the RTK-inhibited-phosphorylation of Akt (Fig. 4C). As internal negative control no significant Akt or Erk phosphorylation was found with FGF or PDGF during sorafenib, EGF during canertinib, and HGF during crizotinib treatment (Fig. 4A, C and D).

### Discrepancies between growth-factor-driven rescue on cell viability level and downstream phosphorylation status

**Fig 5:**
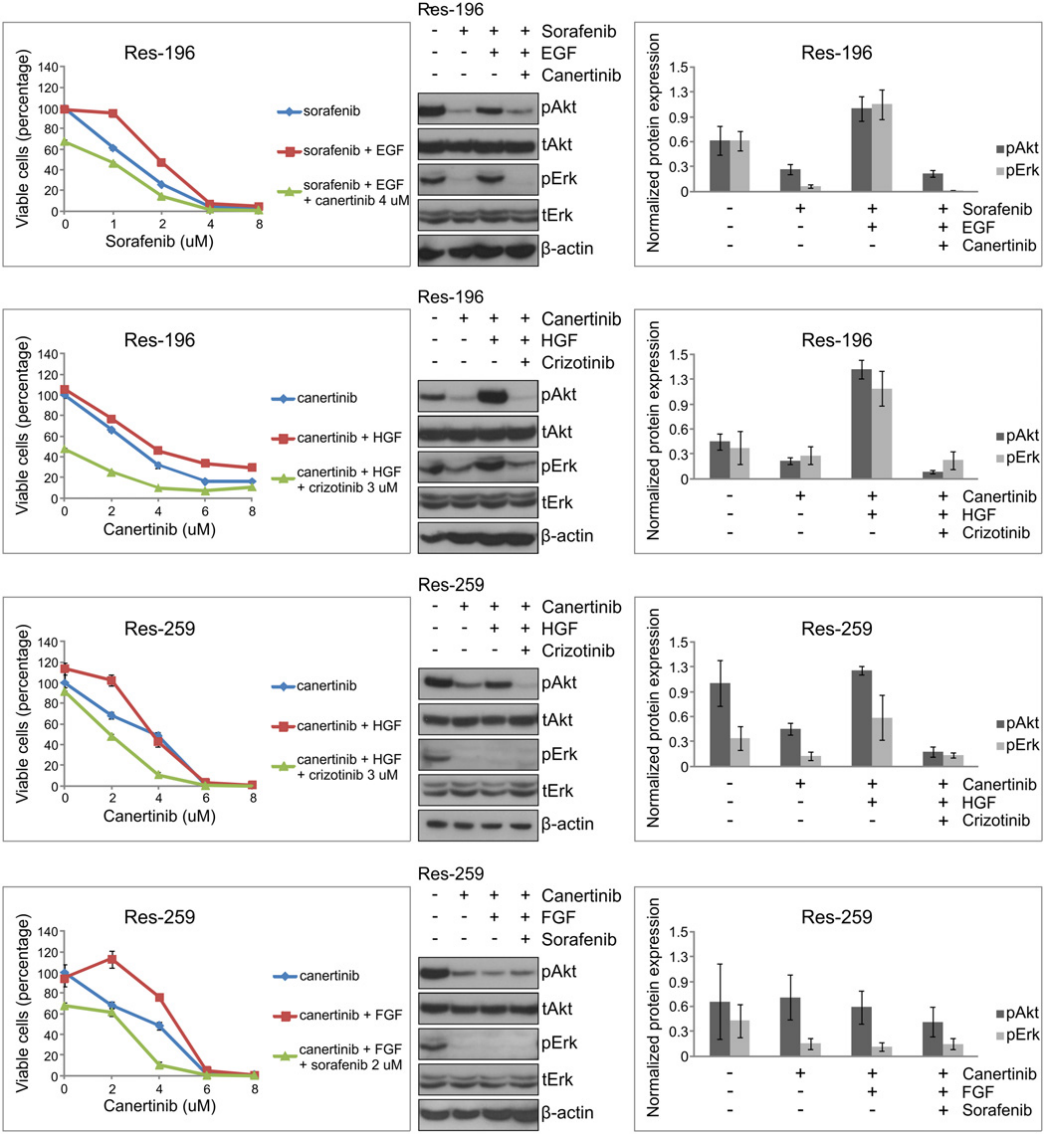
Combined RTK treatment can overcome growth-factor-driven rescue. Cell viability assays demonstrating additional effects of combination treatment in pediatric low grade astrocytoma (Res-259) and ependymoma (Res-196) compared with single treatment (mean ± SD, 48h). Immunoblots showing effect of single RTK inhibition (8 uM), the rescue effect of growth factors (100 ng/ml) and the ability of the second inhibitor (canertinib 4 uM, crizotinib 3 uM, sorafenib 2 uM) to overcome this growth-factor-driven rescue on Akt and Erk phosphorylation (p) in pediatric low grade astrocytoma and ependymoma cells (2h). Protein expression was normalized to β-actin as loading control and derived from three independent experiments of which the mean (± SEM) was calculated. The ability of crizotinib to overcome HGF-driven rescue during canertinib treatment reached statistical significance on Akt and Erk phosphorylation (p = 0.028 and p = 0.046 respectively).

Notably, is the apparent discrepancy of HGF-driven phosphorylation by Akt and Erk (Fig. 5) without increased cell viability during canertinib in Res-259 cells (Fig. 3C). There was HGF-driven rescue observed on cell viability level however at a lower dose than the LC50 and therefore not found in the growth-factor-driven rescue heatmap of Fig. 3C. Also, in dasatinib treated Res-186 cells, HGF was unable to rescue cells at cell viability level despite the expression of the RTK and observed phosphorylation of Akt and Erk. This could possibly be explained by the various downstream targets of dasatinib with the consequence that activation of Akt and Erk is insufficient for cell viability rescue. Vice versa, FGF-driven rescue was not confirmed with Akt or Erk phosphorylation possibly due to involvement of other downstream pathways (Fig. 1), that were not evaluated in this study.

### Combined RTK treatment can overcome growth-factor-driven rescue

**Fig 6:**
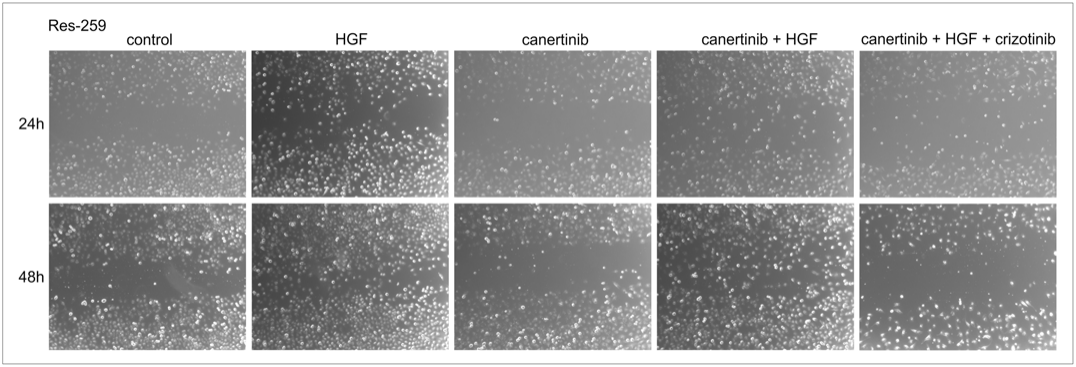
Tumor cell migration. Cell monolayers were scratched with a pipette tip to produce a clear cell-free area into which cells at the periphery could migrate. Micrographs showing migrated Res-259 cells treated with DMSO (control), the effect on tumor cell migration of HGF (100 ng/ml), canertinib (2 uM), HGF addition to canertinib and the combination treatment including canertinib and crizotinib (3 uM) after 24h (upper panels) and 48h (lower panels).

Interestingly, EGF-driven rescue during sorafenib and HGF- and FGF-driven rescue during canertinib could be overcome by combining specific TK inhibitors. As expected, combined RTK treatment in absence of added growth factors resulted in less viable tumor cells than in presence of added growth factors (data not shown). By addition of crizotinib to canertinib treatment, HGF-driven rescue could be overcome and tumor cell survival was disrupted. Moreover, significantly less phosphorylation of Akt (p = 0.028) and Erk (p = 0.046) was observed after combination treatment in Res-196 and Res-259 cells (Fig. 5). Furthermore decrease of apoptotic cells after HGF addition to canertinib 2 uM in Res-259 cells and after EGF addition to sorafenib 1 uM in Res-196 cells, could also be exceeded by crizotinib or canertinib respectively (61,5% and 36.9% more apoptotic tumor cells). In turn, sorafenib can overcome FGF-driven rescue during canertinib treatment (Fig. 5). Actually as rescue by different growth factors was found during sorafenib, including both EGF and HGF, more than one RTK inhibitor would have to be added. Not only on cell survival but also on cell migration EGF-, HGF- and FGF-driven rescue was found. In Fig. 6 the results of canertinib combined with HGF and crizotinib are shown.

In addition, to understand more about this shown growth factor rescue we analyzed growth factor production differences in the absence or presence of sorafenib, canertinib or crizotinib and can conclude that the growth factor production of cultured tumor cells does not differ upon the addition of one of these mentioned RTK inhibitors (S1 Fig.). Moreover, we investigated the expression of the previously described RTKs in the tumor cells treated with or without sorafenib, canertinib or crizotinib and demonstrated that no differences were observed in the percentages of cells expressing RTKs upon inhibitor addition compared to no inhibitor (Res-259 is shown as example in S2 Fig.).

## Discussion

This study showed growth-factor-driven rescue to various RTK inhibitors in pediatric low grade astrocytoma and ependymoma cell lines. Previously, we described several potential interesting therapeutic targets upon kinome profiling in vitro *e*.*g*. VEGFR-2, PDGFRβ, Src, ErbB family members, and HGFR/cMet.[5] In the present study drugs such as sorafenib, dasatinib, canertinib and crizotinib respectively were used to inhibit these targets. In vitro cell viability decreased upon the single use of one of these inhibitors as expected. However, in normal developing brain and therefore, also in the brain tumor (microenvironment) several growth factors including VEGF, EGF, HGF, FGF and PDGF, are commonly expressed.[31–38] Interestingly, the growth factor receptors are found often to be present on low grade astrocytoma and ependymoma cells.

Therefore, the present study investigated the rescue of ligand exposure to these RTK inhibitors. EGF and HGF showed to be effective rescue inducing growth factors in these tumor escape mechanisms acting via their RTKs resulting in increased cell survival and downstream signaling. Interestingly, growth-factor-driven rescue measured during RTK inhibition, was even more pronounced than the results of growth factors on cell survival in the absence of an inhibitor. This phenomenon might be due to a stronger response in a severe stress situation compared to more optimal culture settings. In vitro no changes in growth factor release or RTK expression of the cultured tumor cells was found upon inhibitor addition. Combination treatment showed enhanced decrease in viable tumor cells although a growth factor was present. In ependymoma cells the combination of canertinib and crizotinib showed the best potential strategy, whereas in low grade astrocytoma cells the combination of both canertinib and crizotinib or canertinib and sorafenib demonstrated the best results. These findings highlight the potential growth-factor-induced crosstalk within RTKs that are co-expressed on these pediatric low grade astrocytoma and ependymoma cells.

This is the first study to describe a RTK profile in relation to ligand exposure in low grade astrocytoma. In pediatric ependymoma a positive correlation between higher co-expression of ErbB2 and ErbB4 and tumor proliferative activity has been described.[39] Furthermore, ligand-dependent activation of ErbB receptor-signaling in cultured ependymoma cells resulted in Akt phosphorylation and cellular proliferation that was significantly blocked in a dose-dependent manner using WAY-177820, an inhibitor of ErbB2 tyrosine kinase activity.[39] The present study showed especially EGFR expression compared with the other ErbB family members. Moreover, EGF was shown to be a potential ligand to overcome resistance during other RTK inhibitors in both low grade astrocytoma and ependymoma.

A limitation of the present study is that a certain amount of growth factor was used whereas the exact amount present for interference with RTKs on a cellular level, is still unknown. We are not aware of a study measuring growth factor’s exact availability on the cellular level and therefore we used a dose as used previously by others.[22,24] By doing so, we were able to study the interference of environmental growth factors on tumor cell survival during RTK inhibition.

The rescue by growth factors is in line with previous reports in other tumor types. Particularly, HGF/Met-induced resistance to RTK inhibitors is observed in diverse tumor types, including adult glioblastoma.[40] Further, PDGF upregulation in reaction to VEGFR inhibition and increased FGF/FGFR expression during BRAF or EGFR inhibition may potentially contribute to tumor resistance.[41–44] Recently, tumor-cell-secreted VEGF has been demonstrated as one of the growth-factor-driven rescue mechanisms through activation of VEGFR-2 and the PI3K/Akt pathway.[45] As VEGFR-2 expression is in pediatric low grade astrocytoma mainly limited to endothelial cells,[46] we expect that VEGF will be able to enhance tumor growth only indirectly by inducing angiogenesis. This study underscores the previous result because the cell viability did not differ.

Until now, the first results of single RTK targeted therapy are disappointing in various tumor types due to tumor resistance mechanisms. For pediatric brain tumors, especially low grade astrocytoma and ependymoma only limited studies have been published.[16] ErbB TK inhibitors combined with radiotherapy failed to show increase in progression free survival or overall survival in malignant pediatric brain tumors.[10] Canertinib, inhibiting all four ErbB family members, has not yet been investigated in (pre)clinical pediatric brain tumor studies. Currently, sorafenib is analyzed in pediatric low grade astrocytoma. Although this could be interesting as alterations affecting the BRAF oncogene represent the main genetic defects in pilocytic astrocytoma (WHO grade I),[47,48] we showed growth-factor-driven rescue during sorafenib in pilocytic astrocytoma cells, suggesting less potential for single application of this RTK inhibitor. Crizotinib has been introduced recently in pediatric patients with refractory solid tumors, showing to be well tolerated.[15] A combination of crizotinib and dasatinib is still under investigation in a pediatric brain tumor clinical trial.

As EGFR and HGFR were highly activated in pediatric brain tumors[5] and subsequently high percentages of pediatric brain tumor cells expressed these RTKs, these findings could provide a rational explanation of the limited efficacy of single targeted treatment in clinical trials. Moreover the successful combination treatments to overcome these tumor escape mechanisms support the importance for multi targeted therapy in low grade astrocytoma and ependymoma. Therefore, it is also crucial to take into account the doses that can be achieved without causing high toxicity and the ability of the RTK inhibitors to cross the blood brain barrier, although the presence of an intact or leaky blood brain barrier in children with pediatric brain tumors is still under debate. Coadministration of elacridar could substantially increase dasatinib and crizotinib oral availability and delivery to the brain.[49,50] More interestingly is the finding that canertinib could also increase brain accumulation of another RTK inhibitor in vivo.[51] Our study highlights the extensive importance of environmentally present growth factors in developing escape mechanisms towards RTK inhibitors. It is of great importance to anticipate upon these results for the design of new therapeutic trials with RTK inhibitors in pediatric low grade astrocytomas and ependymomas.

## Supporting Information

S1 Fig Growth factor release in absence or presence of a RTK inhibitor. Human growth factor antibody arrays showing no differences in growth factor release by pediatric low grade astrocytoma (Res-259) and ependymoma cells (Res-196) in absence (DMSO) or presence of a RTK inhibitor (sorafenib, canertinib, crizotinib, LC50, 24h). Bars represent mean signal intensity minus background of the measured spot on the array (± SD).
(TIF)

S2 Fig RTK expression in absence or presence of a RTK inhibitor. Scatter plots showing no differences in percentages of pediatric low grade astrocytoma cells (Res-259) expressing RTKs without (DMSO) or with inhibitor treatment (sorafenib, canertinib, crizotinib, LC50, 24h) using flow cytometry analyses.
(TIF)

## References

[1] Louis DN, Ohgaki H, Wiestler OD, Cavenee WK (2007) Classification of tumours of the central nervous system. Lyon: IARC. 10.1007/s00401-007-0243-4 PMC1929165 17618441

[2] Due-Tonnessen BJ, Helseth E, Scheie D, Skullerud K, Aamodt G, Lundar T (2002) Long-term outcome after resection of benign cerebellar astrocytomas in children and young adults (0–19 years): report of 110 consecutive cases. Pediatr Neurosurg 37: 71–80. 12145515 10.1159/000065108

[3] Fernandez C, Figarella-Branger D, Girard N, Bouvier-Labit C, Gouvernet J, Paz Paredes A, et al (2003) Pilocytic astrocytomas in children: prognostic factors—a retrospective study of 80 cases. Neurosurgery 53: 544–53; discussion 554–5. 12943571 10.1227/01.neu.0000079330.01541.6e

[4] Shim KW, Kim DS, Choi JU (2009) The history of ependymoma management. Childs Nerv Syst 25: 1167–1183. 10.1007/s00381-009-0900-0 19458954

[5] Sikkema AH, Diks SH, den Dunnen WF, ter Elst A, Scherpen FJ, Hoving EW, et al (2009) Kinome profiling in pediatric brain tumors as a new approach for target discovery. Cancer Res 69: 5987–5995. 10.1158/0008-5472.CAN-08-3660 19567681

[6] Jakacki RI, Hamilton M, Gilbertson RJ, Blaney SM, Tersak J, Krailo MD, et al (2008) Pediatric phase I and pharmacokinetic study of erlotinib followed by the combination of erlotinib and temozolomide: a Children's Oncology Group Phase I Consortium Study. J Clin Oncol 26: 4921–4927. 10.1200/JCO.2007.15.2306 18794549 PMC2652086

[7] Broniscer A, Baker SJ, Stewart CF, Merchant TE, Laningham FH, Schaiquevich P, et al (2009) Phase I and pharmacokinetic studies of erlotinib administered concurrently with radiotherapy for children, adolescents, and young adults with high-grade glioma. Clin Cancer Res 15: 701–707. 10.1158/1078-0432.CCR-08-1923 19147777 PMC2629527

[8] Geoerger B, Hargrave D, Thomas F, Ndiaye A, Frappaz D, Andreiuolo F, et al (2011) Innovative Therapies for Children with Cancer pediatric phase I study of erlotinib in brainstem glioma and relapsing/refractory brain tumors. Neuro Oncol 13: 109–118. 10.1093/neuonc/noq141 20974795 PMC3018917

[9] Geyer JR, Stewart CF, Kocak M, Broniscer A, Phillips P, Douglas JG, et al (2010) A phase I and biology study of gefitinib and radiation in children with newly diagnosed brain stem gliomas or supratentorial malignant gliomas. Eur J Cancer 46: 3287–3293. 10.1016/j.ejca.2010.07.005 20708924 PMC2988095

[10] Pollack IF, Stewart CF, Kocak M, Poussaint TY, Broniscer A, Banerjee A, et al (2011) A phase II study of gefitinib and irradiation in children with newly diagnosed brainstem gliomas: a report from the Pediatric Brain Tumor Consortium. Neuro Oncol 13: 290–297. 10.1093/neuonc/noq199 21292687 PMC3064605

[11] Slichenmyer WJ, Elliott WL, Fry DW (2001) CI-1033, a pan-erbB tyrosine kinase inhibitor. Semin Oncol 28: 80–85. 11706399 10.1016/s0093-7754(01)90285-4

[12] Lee EQ, Kuhn J, Lamborn KR, Abrey L, DeAngelis LM, Lieberman F, et al (2012) Phase I/II study of sorafenib in combination with temsirolimus for recurrent glioblastoma or gliosarcoma: North American Brain Tumor Consortium study 05–02. Neuro Oncol 14: 1511–1518. 10.1093/neuonc/nos264 23099651 PMC3499017

[13] Hainsworth JD, Ervin T, Friedman E, Priego V, Murphy PB, Clark BL, et al (2010) Concurrent radiotherapy and temozolomide followed by temozolomide and sorafenib in the first-line treatment of patients with glioblastoma multiforme. Cancer 116: 3663–3669. 10.1002/cncr.25275 20564147

[14] Reardon DA, Vredenburgh JJ, Desjardins A, Peters K, Gururangan S, Sampson JH, et al (2011) Effect of CYP3A-inducing anti-epileptics on sorafenib exposure: results of a phase II study of sorafenib plus daily temozolomide in adults with recurrent glioblastoma. J Neurooncol 101: 57–66. 10.1007/s11060-010-0217-6 20443129 PMC3102498

[15] Mosse YP, Lim MS, Voss SD, Wilner K, Ruffner K, Laliberte J, et al (2013) Safety and activity of crizotinib for paediatric patients with refractory solid tumours or anaplastic large-cell lymphoma: a Children's Oncology Group phase 1 consortium study. Lancet Oncol 14: 472–480. 10.1016/S1470-2045(13)70095-0 23598171 PMC3730818

[16] Sie M, den Dunnen WF, Hoving EW, de Bont ES (2014) Anti-angiogenic therapy in pediatric brain tumors: an effective strategy? Crit Rev Oncol Hematol 89: 418–432. 10.1016/j.critrevonc.2013.09.005 24169416

[17] Maekawa T, Ashihara E, Kimura S (2007) The Bcr-Abl tyrosine kinase inhibitor imatinib and promising new agents against Philadelphia chromosome-positive leukemias. Int J Clin Oncol 12: 327–340. 17929114 10.1007/s10147-007-0699-1

[18] Duncan JS, Whittle MC, Nakamura K, Abell AN, Midland AA, Zawistowski JS, et al (2012) Dynamic reprogramming of the kinome in response to targeted MEK inhibition in triple-negative breast cancer. Cell 149: 307–321. 10.1016/j.cell.2012.02.053 22500798 PMC3328787

[19] Young A, Lou D, McCormick F (2013) Oncogenic and wild-type Ras play divergent roles in the regulation of mitogen-activated protein kinase signaling. Cancer Discov 3: 112–123. 10.1158/2159-8290.CD-12-0231 23103856

[20] Casanovas O, Hicklin DJ, Bergers G, Hanahan D (2005) Drug resistance by evasion of antiangiogenic targeting of VEGF signaling in late-stage pancreatic islet tumors. Cancer Cell 8: 299–309. 16226705 10.1016/j.ccr.2005.09.005

[21] Batchelor TT, Sorensen AG, di Tomaso E, Zhang WT, Duda DG, Cohen KS, et al (2007) AZD2171, a pan-VEGF receptor tyrosine kinase inhibitor, normalizes tumor vasculature and alleviates edema in glioblastoma patients. Cancer Cell 11: 83–95. 17222792 10.1016/j.ccr.2006.11.021 PMC2748664

[22] Wilson TR, Fridlyand J, Yan Y, Penuel E, Burton L, Chan E, et al (2012) Widespread potential for growth-factor-driven resistance to anticancer kinase inhibitors. Nature 487: 505–509. 10.1038/nature11249 22763448 PMC3724525

[23] Bobola MS, Silber JR, Ellenbogen RG, Geyer JR, Blank A, Goff RD (2005) O6-methylguanine-DNA methyltransferase, O6-benzylguanine, and resistance to clinical alkylators in pediatric primary brain tumor cell lines. Clin Cancer Res 11: 2747–2755. 15814657 10.1158/1078-0432.CCR-04-2045

[24] Omerovic J, Hammond DE, Clague MJ, Prior IA (2008) Ras isoform abundance and signalling in human cancer cell lines. Oncogene 27: 2754–2762. 17998936 10.1038/sj.onc.1210925 PMC2557550

[25] Ramakrishnan V, Timm M, Haug JL, Kimlinger TK, Halling T, Wellik LE, et al (2012) Sorafenib, a multikinase inhibitor, is effective in vitro against non-Hodgkin lymphoma and synergizes with the mTOR inhibitor rapamycin. Am J Hematol 87: 277–283. 10.1002/ajh.22263 22190165 PMC3465673

[26] Jane EP, Premkumar DR, Pollack IF (2006) Coadministration of sorafenib with rottlerin potently inhibits cell proliferation and migration in human malignant glioma cells. J Pharmacol Exp Ther 319: 1070–1080. 16959960 10.1124/jpet.106.108621

[27] Chapuy B, Schuelper N, Panse M, Dohm A, Hand E, Schroers R, et al (2011) Multikinase inhibitor sorafenib exerts cytocidal efficacy against Non-Hodgkin lymphomas associated with inhibition of MAPK14 and AKT phosphorylation. Br J Haematol 152: 401–412. 10.1111/j.1365-2141.2010.08526.x 21689083

[28] Sugiyama H, Onuki K, Ishige K, Baba N, Ueda T, Matsuda S, et al (2011) Potent in vitro and in vivo antitumor activity of sorafenib against human intrahepatic cholangiocarcinoma cells. J Gastroenterol 46: 779–789. 10.1007/s00535-011-0380-3 21331764

[29] Djerf Severinsson EA, Trinks C, Green H, Abdiu A, Hallbeck AL, Stal O, et al (2011) The pan-ErbB receptor tyrosine kinase inhibitor canertinib promotes apoptosis of malignant melanoma in vitro and displays anti-tumor activity in vivo. Biochem Biophys Res Commun 414: 563–568. 10.1016/j.bbrc.2011.09.118 21982771

[30] Shao H, Peng T, Ji Z, Su J, Zhou X (2013) Systematically studying kinase inhibitor induced signaling network signatures by integrating both therapeutic and side effects. PLoS One 8: e80832 10.1371/journal.pone.0080832 24339888 PMC3855094

[31] Lafuente JV, Ortuzar N, Bengoetxea H, Bulnes S, Argandona EG (2012) Vascular endothelial growth factor and other angioglioneurins: key molecules in brain development and restoration. Int Rev Neurobiol 102: 317–346. 10.1016/B978-0-12-386986-9.00012-0 22748835

[32] Namba H, Zheng Y, Abe Y, Nawa H (2009) Epidermal growth factor administered in the periphery influences excitatory synaptic inputs onto midbrain dopaminergic neurons in postnatal mice. Neuroscience 158: 1731–1741. 10.1016/j.neuroscience.2008.10.057 19041374

[33] Rosser AE, Tyers P, Dunnett SB (2000) The morphological development of neurons derived from EGF- and FGF-2-driven human CNS precursors depends on their site of integration in the neonatal rat brain. Eur J Neurosci 12: 2405–2413. 10947819 10.1046/j.1460-9568.2000.00135.x

[34] Achim CL, Katyal S, Wiley CA, Shiratori M, Wang G, Oshika E, et al (1997) Expression of HGF and cMet in the developing and adult brain. Brain Res Dev Brain Res 102: 299–303. 9352114 10.1016/s0165-3806(97)00108-9

[35] Thewke DP, Seeds NW (1999) The expression of mRNAs for hepatocyte growth factor/scatter factor, its receptor c-met, and one of its activators tissue-type plasminogen activator show a systematic relationship in the developing and adult cerebral cortex and hippocampus. Brain Res 821: 356–367. 10064822 10.1016/s0006-8993(99)01115-4

[36] Gremo F, Presta M (2000) Role of fibroblast growth factor-2 in human brain: a focus on development. Int J Dev Neurosci 18: 271–279. 10715581 10.1016/s0736-5748(99)00095-7

[37] Valenzuela CF, Kazlauskas A, Weiner JL (1997) Roles of platelet-derived growth factor in the developing and mature nervous systems. Brain Res Brain Res Rev 24: 77–89. 9233543 10.1016/s0165-0173(97)00012-x

[38] Jain RK, di Tomaso E, Duda DG, Loeffler JS, Sorensen AG, Batchelor TT (2007) Angiogenesis in brain tumours. Nat Rev Neurosci 8: 610–622. 17643088 10.1038/nrn2175

[39] Gilbertson RJ, Bentley L, Hernan R, Junttila TT, Frank AJ, Haapasalo H, et al (2002) ERBB receptor signaling promotes ependymoma cell proliferation and represents a potential novel therapeutic target for this disease. Clin Cancer Res 8: 3054–3064. 12374672

[40] Jun HJ, Acquaviva J, Chi D, Lessard J, Zhu H, Woolfenden S, et al (2012) Acquired MET expression confers resistance to EGFR inhibition in a mouse model of glioblastoma multiforme. Oncogene 31: 3039–3050. 10.1038/onc.2011.474 22020333 PMC3774279

[41] Ho AL, Vasudeva SD, Lae M, Saito T, Barbashina V, Antonescu CR, et al (2012) PDGF receptor alpha is an alternative mediator of rapamycin-induced Akt activation: implications for combination targeted therapy of synovial sarcoma. Cancer Res 72: 4515–4525. 10.1158/0008-5472.CAN-12-1319 22787122 PMC3432680

[42] di Tomaso E, Snuderl M, Kamoun WS, Duda DG, Auluck PK, Fazlollahi L, et al (2011) Glioblastoma recurrence after cediranib therapy in patients: lack of "rebound" revascularization as mode of escape. Cancer Res 71: 19–28. 10.1158/0008-5472.CAN-10-2602 21199795 PMC3074948

[43] Ware KE, Marshall ME, Heasley LR, Marek L, Hinz TK, Hercule P, et al (2010) Rapidly acquired resistance to EGFR tyrosine kinase inhibitors in NSCLC cell lines through de-repression of FGFR2 and FGFR3 expression. PLoS One 5: e14117 10.1371/journal.pone.0014117 21152424 PMC2994708

[44] Yadav V, Zhang X, Liu J, Estrem S, Li S, Gong XQ, et al (2012) Reactivation of mitogen-activated protein kinase (MAPK) pathway by FGF receptor 3 (FGFR3)/Ras mediates resistance to vemurafenib in human B-RAF V600E mutant melanoma. J Biol Chem 287: 28087–28098. 10.1074/jbc.M112.377218 22730329 PMC3431627

[45] Akiyama K, Ohga N, Hida Y, Kawamoto T, Sadamoto Y, Ishikawa S, et al (2012) Tumor endothelial cells acquire drug resistance by MDR1 up-regulation via VEGF signaling in tumor microenvironment. Am J Pathol 180: 1283–1293. 10.1016/j.ajpath.2011.11.029 22245726

[46] Sikkema AH, de Bont ES, Molema G, Dimberg A, Zwiers PJ, Diks SH, et al (2011) Vascular endothelial growth factor receptor 2 (VEGFR-2) signalling activity in paediatric pilocytic astrocytoma is restricted to tumour endothelial cells. Neuropathol Appl Neurobiol 37: 538–548. 10.1111/j.1365-2990.2011.01160.x 21208252

[47] Gronych J, Korshunov A, Bageritz J, Milde T, Jugold M, Hambardzumyan D, et al (2011) An activated mutant BRAF kinase domain is sufficient to induce pilocytic astrocytoma in mice. J Clin Invest 121: 1344–1348. 10.1172/JCI44656 21403401 PMC3069779

[48] Rodriguez FJ, Lim KS, Bowers D, Eberhart CG (2013) Pathological and molecular advances in pediatric low-grade astrocytoma. Annu Rev Pathol 8: 361–379. 10.1146/annurev-pathol-020712-164009 23121055 PMC3600584

[49] Lagas JS, van Waterschoot RA, van Tilburg VA, Hillebrand MJ, Lankheet N, Rosing H, et al (2009) Brain accumulation of dasatinib is restricted by P-glycoprotein (ABCB1) and breast cancer resistance protein (ABCG2) and can be enhanced by elacridar treatment. Clin Cancer Res 15: 2344–2351. 10.1158/1078-0432.CCR-08-2253 19276246

[50] Chuan Tang S, Nguyen LN, Sparidans RW, Wagenaar E, Beijnen JH, Schinkel AH (2014) Increased oral availability and brain accumulation of the ALK inhibitor crizotinib by coadministration of the P-glycoprotein (ABCB1) and breast cancer resistance protein (ABCG2) inhibitor elacridar. Int J Cancer 134: 1484–1494. 10.1002/ijc.28475 24037730

[51] Minocha M, Khurana V, Qin B, Pal D, Mitra AK (2012) Enhanced brain accumulation of pazopanib by modulating P-gp and Bcrp1 mediated efflux with canertinib or erlotinib. Int J Pharm 436: 127–134. 10.1016/j.ijpharm.2012.05.038 22688250 PMC3573846

