# Supplementary figures and images for: Growth-Factor-Driven Rescue to Receptor Tyrosine Kinase (RTK) Inhibitors through Akt and Erk Phosphorylation in Pediatric Low Grade Astrocytoma and Ependymoma
Source: PLoS One. 2015 Mar 23;10(3):e0122555. doi: 10.1371/journal.pone.0122555 (PMC4370756; doi:10.1371/journal.pone.0122555)

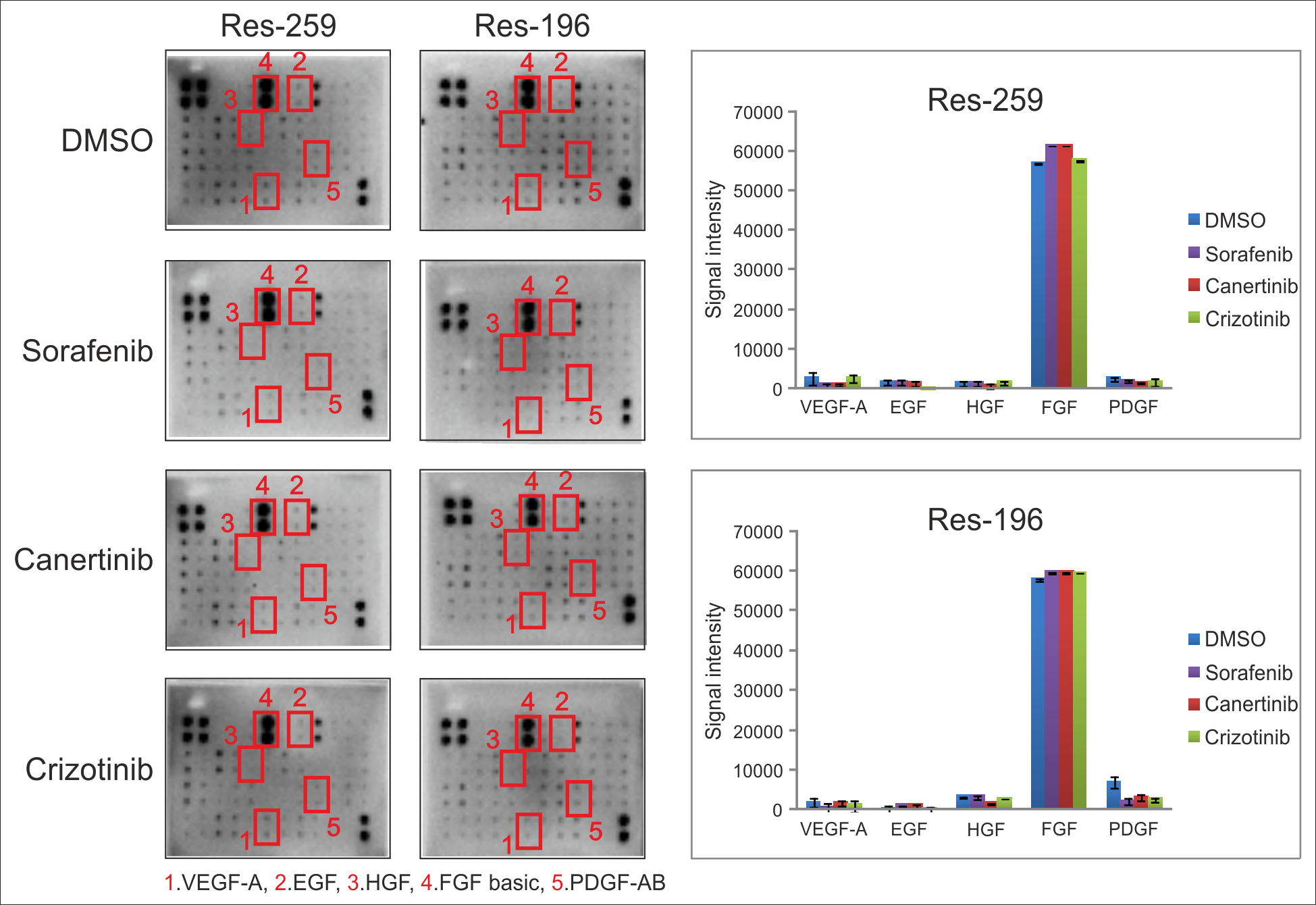

Supplement: S1 Fig — Human growth factor antibody arrays showing no differences in growth factor release by pediatric low grade astrocytoma (Res-259) and ependymoma cells (Res-196) in absence (DMSO) or presence of a RTK inhibitor (sorafenib, canertinib, crizotinib, LC50, 24h). Bars represent mean signal intensity minus background of the measured spot on the array (± SD). (TIF) [file pone.0122555.s001.tif]

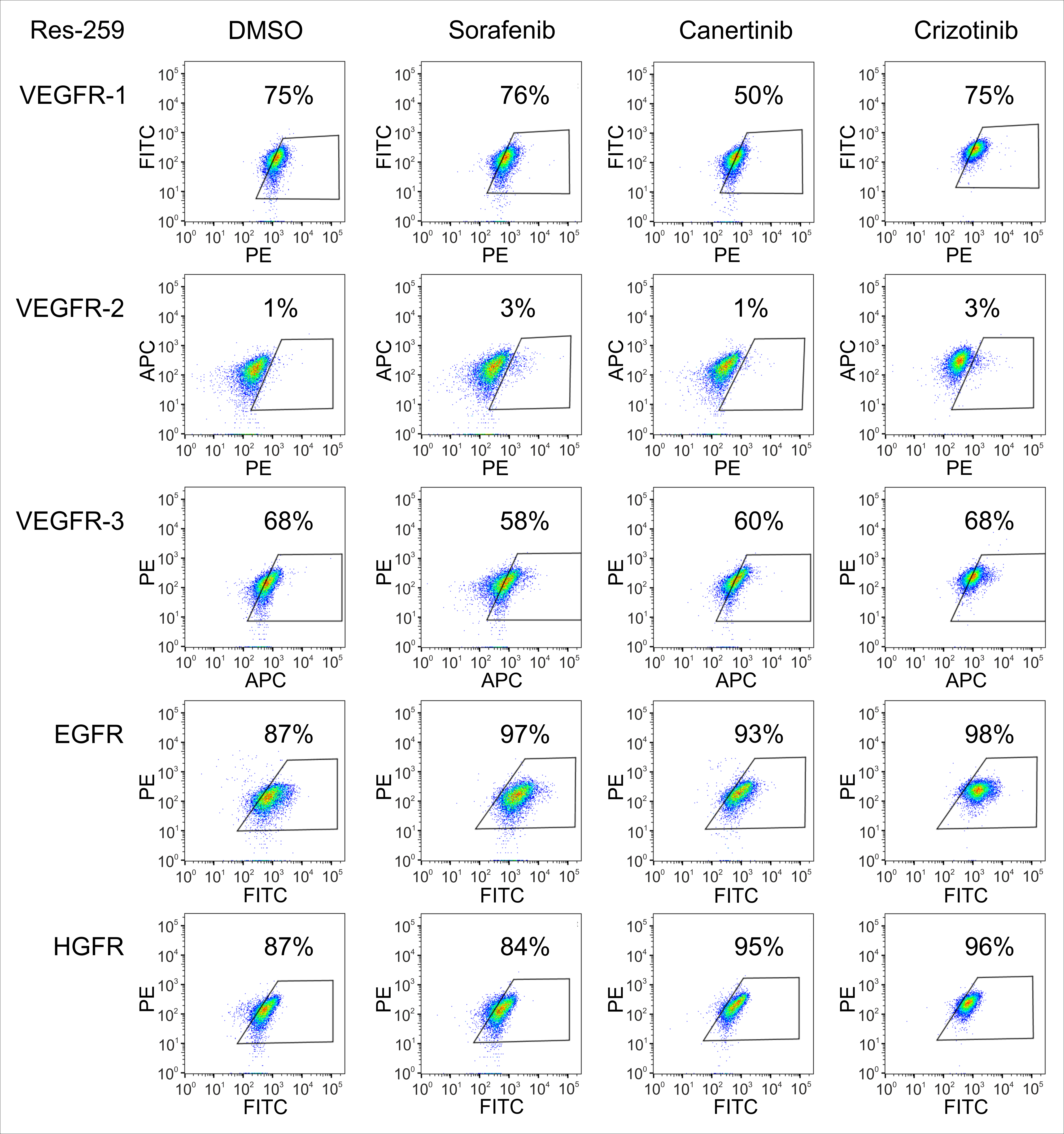

Supplement: S2 Fig — Scatter plots showing no differences in percentages of pediatric low grade astrocytoma cells (Res-259) expressing RTKs without (DMSO) or with inhibitor treatment (sorafenib, canertinib, crizotinib, LC50, 24h) using flow cytometry analyses. (TIF) [file pone.0122555.s002.tif]
